# Associations among serum pro- and anti-inflammatory cytokines, metabolic mediators, body condition, and uterine disease in postpartum dairy cows

**DOI:** 10.1186/1477-7827-11-103

**Published:** 2013-11-09

**Authors:** Ramanathan K Kasimanickam, Vanmathy R Kasimanickam, Jesse R Olsen, Erin J Jeffress, Dale A Moore, John P Kastelic

**Affiliations:** 1 Department of Veterinary Clinical Sciences, Washington State University, Pullman, WA 99164, USA; 2 Center for Veterinary and Health Sciences, Oklahoma State University, Stillwater, OK 74078, USA; 3 Department of Production Animal Health, University of Calgary, Calgary AB T2N 4N1, Canada

**Keywords:** Adipokines, Insulin, IGF-1, Postpartum, Uterine inflammation, Body condition, Dairy cows

## Abstract

**Background:**

Adipose tissue is an active endocrine organ which secretes a wide range of hormones and protein factors, collectively termed adipokines. Adipokines affect appetite and satiety, glucose and lipid metabolism, inflammation and immune functions. The objectives were to evaluate serum concentrations of adipokines (adiponectin, leptin, tumor necrosis factor (TNF)-alpha, interleukin (IL)-1beta and IL-6) in lactating dairy cows with postpartum uterine inflammatory conditions (metritis, clinical endometritis or subclinical endometritis) and in cows experiencing loss of body condition, and to assess the relationship of adipokines and body condition loss in the establishment of persistent uterine inflammatory conditions.

**Methods:**

Lactating multiparous Holstein cows (N = 40), with body condition scores (BCS) from 2 to 4 (eight cows for each 0.5 score increment) were enrolled. Body condition was monitored for all cows weekly for 7 weeks post calving; cows with uterine inflammatory conditions were also re-evaluated 2 weeks later. Blood samples were collected from 1 week prior to calving to 7 weeks after calving for determination of serum concentrations of adipokines, insulin and insulin like growth factor (IGF)-1.

**Results:**

Cows with metritis or clinical endometritis had higher serum concentrations of adiponectin, leptin, TNF-alpha, IL-1beta and IL-6 compared to normal cows (*P* < 0.05). Furthermore, serum leptin, TNF-alpha, IL-1beta and IL-6 were higher in cows with subclinical endometritis compared to normal cows (*P* < 0.05), and insulin and IGF-1 concentrations were lower in cows with metritis or clinical endometritis. Cows with low BCS (2 and 2.5) had significantly higher adiponectin, TNF-alpha, IL-1beta and IL-6 than those with high BCS (3 to 4). Cows with persistent uterine inflammatory conditions had higher adiponectin, leptin TNF-alpha, IL-1beta and IL-6 and insulin compared to normal and spontaneously recovered cows, except for IGF-1 (P < 0.05).

**Conclusions:**

Serum concentrations of adipokines, insulin, and IGF-1 had significant associations with BCS categories (low vs. high) and postpartum uterine inflammatory conditions. Perhaps loss of body condition mediated increases in anti- and pro-inflammatory cytokines, whereas increased pro- and anti-inflammatory cytokines concentrations mediated body condition loss and thereby prolonged persistence of uterine inflammation in dairy cows.

## Background

Postpartum uterine inflammatory conditions in dairy cows are of great economic importance due to their adverse effects on reproductive performance and subsequent milk production [1]. Clinical and subclinical endometritis are common in high-producing dairy cattle, damaging the endometrium, delaying the onset of ovarian cyclic activity after calving, extending luteal phases, and reducing fertility [2-5]. During the periparturient period, several aspects of the bovine immune system change; however, cows that fail to limit infection are predisposed to uterine disease [6-8]. The initial defense of the endometrium against uterine pathogens is the innate immune system [6-8]; cytokines and chemokines direct the influx of neutrophils into the uterus [6-8]. However, the functional capacity of neutrophils is reduced after calving in many dairy cows, predisposing them to establishment of a uterine infection [9].

Body condition loss in dairy cows during the periparturient period is associated with several postpartum disorders, including uterine infections [9-12]. Cytokines and/or cytokine-mediated neural and endocrine changes have key roles in rapid and intensive loss of body condition and weight [13]. Perhaps uterine infections and resulting metabolic and endocrine changes predispose cows to lose body condition.

Adipose tissue is an active endocrine organ that communicates with the brain and peripheral tissues by secreting a wide range of hormones and protein factors, collectively termed adipokines [14,15]. These factors include adiponectin, leptin, cytokines [tumor necrosis factor (TNF)-α, interleukins (IL)], chemokines, acute phase proteins, homeostatic and hemodynamic factors and neurotrophins [14,15]. Adiponectin possesses both anti-inflammatory and anti-obesity effects, whereas TNF-α and IL-6 are considered pro-inflammatory cytokines [16-18]. Adiponectin plays an important role in energy homeostasis, with involvement in regulating glucose concentrations through reductions in insulin resistance and fatty acid breakdown, whereas leptin regulates food intake and energy expenditure [16-20]. Leptin regulates food intake as well as metabolic and endocrine functions. Leptin also plays a regulatory role in immunity, inflammation, and hematopoiesis [21]. Alterations in immune and inflammatory responses are present in leptin- or leptin-receptor-deficient animals. Both leptin and its receptor share structural and functional similarities with the interleukin-6 family of cytokines. Leptin exerts proliferative and antiapoptotic activities in a variety of cell types, including T lymphocytes, leukemia cells, and hematopoietic progenitors. Leptin also affects cytokine production, the activation of monocytes/macrophages, wound healing, angiogenesis, and hematopoiesis. Leptin production is mainly regulated by insulin-induced changes of adipocyte metabolism [22]. Adiponectin increases insulin sensitivity, perhaps by increasing tissue fat oxidation resulting in reduced circulating fatty acid levels and reduced intramyocellular or liver triglyceride content. Adiponectin and leptin together normalize insulin action in severely insulin-resistant animals that have very low levels of adiponectin and leptin due to lipoatrophy [22]. Leptin also improves insulin resistance and reduces hyperlipidemia in lipoatrophic humans. Adiponectin production is stimulated by agonists of peroxisome proliferator-activated receptor-gamma, an action that may contribute to the insulin-sensitizing effects of this class of compounds [23]. The production of adiponectin, leptin and insulin is influenced by nutritional status.

The objectives of the current study were to evaluate serum concentrations of adipokines (adiponectin, leptin, TNF-α, IL-1β and IL-6) and metabolic markers (insulin and insulin-like growth factor 1 (IGF-1)), as well as body condition in relation to postpartum, and to detect postpartum uterine inflammatory conditions (metritis, clinical endometritis and subclinical endometritis). We hypothesized that uterine disease increases adipokine concentrations, reduces body condition, and alters metabolic markers, thereby increasing the incidence of persistent uterine inflammatory conditions in postpartum dairy cows.

## Methods

### Animals

Lactating Holstein cows, (N = 40; multiparous with a mean lactation number of 3 (range: from 2 to 6), from a dairy farm in Washington state with no history of peripartum diseases were enrolled in this study. Cows were fed, twice daily a total mixed ration to meet or exceed dietary requirements for lactating Holstein cows weighing 545 to 770 kg and producing 27 to 36 kg of 3.5% fat-corrected milk. Eight cows in each body condition score category from 2 to 4, with 0.5 score increments, were selected for the study. In all cows, body condition scoring (emaciated 1; obese 5) was done once weekly at enrollment from 1 week prior to calving to 7 weeks postcalving [24]. Body condition scores were given by 2 operators. Cows were categorized for BCS as low (2 and 2.5) and high (3, 3.5 and 4). Blood samples were collected (coccygeal venipuncture) weekly, at or before feeding from 1 week prior to expected calving to 7 weeks after calving, for determination of serum concentrations of adipokines (adiponectin, leptin, TNF-α, IL-1β, IL-6), insulin and IGF-1. Cows were monitored during weekly visits up to 7 weeks post calving, for the presence of metritis, clinical endometritis, and subclinical endometritis utilizing diagnostic techniques and criteria as described (Table 1) [3-5,7,25]. Cows were re-evaluated 2 weeks after initial uterine disease diagnosis to determine new cases, spontaneous recovery and/or persistence of uterine inflammatory conditions. No treatments were administered during the study period except that all cows received 1 to 2 uterine lavages in the first week post calving. A cow diagnosed with uterine disease at both initial and follow-up examinations was considered to have persistent inflammation. The incidence of persistent inflammation was calculated as number of cows with uterine disease at both the initial and follow-up examinations, divided by cows that had uterine inflammatory conditions at initial examination. The 40 cows included in this study were designated, based on initial and re-examinations, in one of the following groups: normal, metritis, clinical endometritis, and subclinical endometritis. In addition, for each group, adipokine concentrations were considered retrospectively during the postpartum period.

**Table 1:** Diagnostic criteria of uterine inflammatory conditions used for initial diagnosis and re-evaluation (2 weeks later) in dairy cows

**Uterine inflammatory conditions**	**DIM @ initial diagnosis**	**Diagnostic criteria**	
		**Initial diagnosis**	**Reevaluation§**
*Metritis*	1-14	Foul smelling watery, brown uterine discharge with or without ≥ 39.4 rectal temperature	Mucopurulent uterine discharge or > 18% PMN in endometrial cytology
*Clinical endometritis*	28-35	Mucopurulent uterine discharge	Mucopurulent uterine discharge or > 18% PMN in endometrial cytology
*Subclinical endometritis*	28-35	> 18% PMN in endometrial cytology (No uterine discharge)	> 10% PMN in endometrial cytology (No uterine discharge)

DIM – Days in milk.

PMN – Polymorphonuclear neutrophils.

§Re-evaluation 2 weeks after initial diagnosis.

### Adipokines

Serum adipokine concentrations were determined by ELISA. All primary and secondary antibodies were purchased from Santa Cruz Biotechnology (Santa Cruz, CA, USA), unless otherwise stated. Briefly, 96-well plates were pre-coated with standards and samples for all serum adipokines and kept at 4°C for at least 24 h. For adiponectin determination, 100 μL of affinity-purified goat polyclonal antibody raised against Acrp30 of human origin (sc-26496), recommended for use in cattle were added to the respective 96-well plates. Concentrations of TNF-α were estimated by adding 100 μL of goat polyclonal antibody raised against TNF-α of mouse origin (sc-1348), recommended for use in cattle. Similarly, for leptin, 100 μL of affinity-purified rabbit polyclonal antibody raised against leptin of human origin (sc-842) with a significant cross reactivity to cattle was used. Likewise, IL-1β and IL-6 were separately estimated using 100 μL of affinity-purified rabbit polyclonal antibody raised against bovine IL-1β (ab 23778, Abcam, Cambridge, MA, USA), and ovine IL-6 with cross reactivity against bovine IL-6 (AHP424, Serotec, Raleigh, NC, USA). Corresponding peptides or proteins (sc- 26496 P, sc-1348 P, sc-842 P, ab88013 and IL6-241B) used for antibody production were used to prepare standards at various concentrations. Ranges for standard dilution and serum dilution for each protein target were determined from a pre-run of standards and at least three samples. In short, at least eight dilutions of each sample (1 in 10, 1 in 50, 1 in 100, 1 in 200, 1 in 400, 1 in 600, 1 in 800 and 1 in 1000) were used to make dilution curve and compared to the regression line of the standard [26-28]. Then the serum dilution closest to the regression line of standard was selected. Standard dilution ranges were carefully chosen to include lowest and highest reading of all samples [26-28]. The shape of the regression line and serum dilution used was different for each target. Each 96-well plate had one set of standards and samples in triplicate. The ELISA plates were coated with 100 μL of standards or samples. More than one 96-well plate was used for one target. Then, non-specific protein binding was blocked by adding 150 μL of either 2% donkey or goat serum in PBS to each well (depending on the species used to raise secondary antibodies). The plate was left on a rocking platform at room temperature for 60 min with blocking buffer. Wash buffer was prepared with 0.05% tween20 in PBS. Following non-specific protein blocking, samples were incubated (60 min) with primary antibodies. These antibodies had 200 μg/mL initial concentrations, but were diluted to 1:100 before incubation. After washing with wash buffer, 100 μL of secondary antibodies (60 min), donkey anti-goat IgG-HRP (sc-2020) for adiponectin and TNF-α and goat anti-rabbit IgG-HRP (sc-2030) for leptin, IL-1β and IL-6, respectively, were added to each well. After washing with buffer, 100 μL of reagent containing the substrate of acetyl cholinesterase (N301, Thermo Scientific, Logan, UT, USA) was added for the enzymatic reaction until color development and then 50 μL of stop solution (N600, Thermo Scientific) was added after color development. Plates were read at 450 nm using a Glomax®-Multi Detection System (Promega, Madison, WI, USA) and values of serum adiponectin (ng/mL), leptin (ng/mL), IL-6 (ng/mL), IL1β (ng/mL) and TNF-α (ng/mL) concentrations were calculated from standard curves. Mean readings and dilution factors were used to calculate concentrations. Intra-assay coefficient was determined based on the triplicates of the sample. Inter-assay coefficient was calculated based on replicates performed. Ten samples per target were repeated four times [26-28]. The intra- and inter-assay CVs were 7.0 and 10.6% for adiponectin, 6.8 and 9.8% for leptin, 7.3 and 12.8% for IL-6, 10.2 and 14.3% for IL-1β, and 8.4 and 13.4% for TNF-α, respectively.

### Insulin

Insulin was measured by ELISA (as described above), using affinity-purified mouse monoclonal antibody raised against full-length porcine insulin, which is similar to bovine insulin except one amino acid (sc-8033) and goat anti-mouse IgG-HRP (sc-2062) by the method described above. Human recombinant insulin (sc-29062), which is homologous to bovine insulin, was used to prepare standards. The intra- and inter-assay CVs were 9.2 and 10.5%, respectively.

### Insulin like growth factor 1

For IGF-1, ELISA was performed using affinity purified rabbit polyclonal antibody raised against amino acids of human origin IGF-1 (which is also recommended for detection in cattle; sc-9013) and secondary antibody, goat anti-rabbit IgG-HRP (sc-2030), as described above. For these assays, IGF-1 biologically active peptide of human origin, which is very similar to bovine IGF-1 (sc-4589), was used to prepare standards of known concentrations. The intra- and inter-assay CVs averaged 6.9 and 11.1%, respectively.

### Statistical analyses

The MIXED procedure of the SAS System (SAS version 9.12, SAS Institute, Cary, NC, USA) was used to perform a repeated measures ANOVA to determine effects of uterine inflammatory condition categories (metritis, clinical endometritis, subclinical endometritis) and normal (grouped at the end of the study period), persistent uterine inflammatory condition categories (persistently infected vs. normal and recovered cows), body condition score categories [low (2 and 2.5) vs. high (3, 3.5 and 4)], time from −1 to 7 weeks postpartum, and their interactions, on mean serum adipokines, IGF-1 and insulin concentrations. Additionally, repeated measures ANOVA were performed separately to determine the effects of uterine inflammatory condition, persistent uterine inflammatory condition categories (persistent vs. normal, recovered uterine inflammation), body condition score categories [low (2 and 2.5) vs. high (3, 3.5 and 4)], time from −1 to 7 weeks postpartum, and their interactions on mean serum adipokines, IGF-1 and insulin concentrations. Differences in mean concentrations of adipokines, IGF-1 and insulin within uterine inflammatory conditions and between weeks postpartum intervals, and within week postpartum interval and between uterine inflammatory conditions were tested by creating contrast statements. Goodness of fit of the statistical model (inclusion/exclusion of random effects, variance/covariance structure selection etc.) was evaluated using the Bayesian information criterion (BIC, 857.7 to 1057.9), fit statistic and the restricted log-likelihood function (−2LL, 823.4 to 1100.4), where lower values indicated better fit, and the value and significance of the fixed effect model parameters were tested. All data were evaluated for normality of their distribution using PROC Univariate method. Values were transformed using logarithmic or square-root transformations, but non-transformed data were presented. For all analyses, *P* ≤ 0.05 was considered significant.

Sample size calculations were done *a priori*. A minimum of two cows per group were needed to detect a 0.58 ± 0.22 mean difference in adipokine concentrations between cows with uterine inflammatory conditions, or to detect a 0.21 ± 0.03 mean difference in adipokine concentrations between cows with low versus high BCS categories.

## Results

Of the 40 cows monitored, five (12.5%) were diagnosed with metritis, 16 (40.0%) had clinical endometritis, six (15%) had subclinical endometritis, and the remaining 13 (32.5%) were categorized as normal. However, four “normal” cows [30.8%; (4/13] developed clinical endometritis 2 weeks later (beyond 35 DIM). Furthermore, 18 affected cows [66.7% (18/27)] had a persistent uterine inflammation 2 weeks later. All five cows diagnosed with metritis had clinical endometritis or subclinical endometritis when evaluated 2 weeks later. Five cows (31.3%) with clinical endometritis and four cows (66.7%) with subclinical endometritis recovered spontaneously within 2 weeks. Ten cows with persistent uterine inflammation lost at least 0.5 body condition score compared to eight normal and/or spontaneously recovered cows between 1 and 5 weeks postpartum [55.6% (10/18) vs. 44.4%, (8/18)].

### Serum concentrations of adipokines, insulin, and IGF-1 in uterine inflammatory condition

Serum adipokine concentrations differed among uterine conditions (metritis, clinical endometritis, subclinical endometritis and normal), weeks postpartum [−1, 0, 1 to 7], and body condition scores [2 and 2.5 (low) vs. 3, 3.5 and 4 (high)] (Table 2; P < 0.05). There were significant interactions for the weeks in postpartum by body condition score category, uterine inflammatory condition by weeks in postpartum and for the uterine inflammatory condition by body condition score category (Table 2; P < 0.05).

**Table 2:** ‘P’ values* from repeated measures of ANOVA for the effects of uterine inflammatory conditions‡ and body condition score categories [low (2 and 2.5) and high (3, 3.5 and 4)] and weeks postpartum on adipokines in postpartum dairy cows

**Adipokine**	**Effect**	**DF**	**F Value**	***P*** **> F**
*Adiponectin*	Uterine inflammatory conditions	3	32.45	<0.0001
	Body condition score categories	1	6.09	0.001
	Weeks in postpartum	8	16.52	0.01
*Leptin*	Uterine inflammatory conditions	3	28.44	0.001
	Body condition score categories	1	6.80	0.01
	Weeks in postpartum	8	17.95	0.03
*TNF-α*	Uterine inflammatory conditions	3	24.91	0.0005
	Body condition score categories	1	7.87	0.009
	Weeks in postpartum	8	16.20	0.04
*IL-1β*	Uterine inflammatory conditions	3	26.59	<0.0001
	Body condition score categories	1	6.53	0.001
	Weeks in postpartum	8	17.1	0.02
*IL-6*	Uterine inflammatory conditions	3	29.52	<0.0001
	Body condition score categories	1	7.99	0.01
	Weeks in postpartum	8	13.52	0.01
*Insulin*	Uterine inflammatory conditions	3	25.12	<0.001
	Body condition score categories	1	8.11	0.03
	Weeks in postpartum	8	15.25	0.02
*IGF-1*	Uterine inflammatory conditions	3	23.83	<0.001
	Body condition score categories	1	7.93	0.01
	Weeks in postpartum	8	14.25	0.01

*Accounted for by uterine inflammatory conditions by body condition score categories, uterine inflammatory conditions by weeks in postpartum and body condition score categories by weeks in postpartum interactions (P < 0.001); ‡Refer Table 1 for definition for uterine inflammatory conditions.

Serum adiponectin concentrations at the time of diagnosis were higher in cows with metritis or clinical endometritis compared to those with subclinical endometritis or normal cows (Table 3; P < 0.05). Serum concentrations of TNF-α were higher in cows with metritis, clinical endometritis or subclinical endometritis compared to normal cows (Table 3; P < 0.05). Serum concentrations of leptin and IL-6 were lower in cows diagnosed with subclinical endometritis compared to those with metritis or clinical endometritis (Table 3; P < 0.05). Cows with subclinical endometritis had higher leptin and IL-6 concentrations compared to normal cows (Table 3; P < 0.05), whereas IL-1β concentrations were higher in cows with any diagnosed uterine inflammatory conditions compared to normal cows (Table 3; P < 0.05). The mean serum concentrations reported here are values at the time of diagnosis.

**Table 3:** Mean ± SEM* serum adipokine concentrations in cows with postpartum uterine inflammatory conditions in dairy cows

**Adipokine**	**n**	**Adiponectin (ng/mL)**	**Leptin (ng/mL)**	**TNF-a (ng/mL)**	**IL-1β (ng/mL)**	**IL-6 (ng/mL)**	**Insulin (ng/mL)**	**IGF-1 (ng/mL)**
*Metritis‡*	5	479.8 ± 23.9^a^	6.74 ± 0.34^a^	2.21 ± 0.11^a^	2.83 ± 0.14^a^	3.23 ± 0.16^a^	0.28 ± 0.01	135 ± 9.1
*Clinical endometritis§*	16	470 ± 23.5^a^	6.42 ± 0.32^a^	1.99 ± 0.10^ab^	3.01 ± 0.15^a^	3.34 ± 0.17^a^	0.57 ±0.04	132 ± 9.3
*Subclinical endometritis§*	6	412 ± 20.6^b^	4.64 ± 0.26^b^	1.63 ± 0.08^b^	3.42 ± 0.17^a^	2.58 ± 0.13^b^	0.42 ± 0.02	192 ± 12.1
*Normal§*	13	428 ± 21.4^b^	3.91 ± 0.20^c^	0.68 ± 0.03^c^	1.51 ± 0.08^b^	1.72 ± 0.09^c^	0.45 ± 0.03	202 ± 16.3

‡ Mean ± SEM serum concentrations of adiponectin, leptin, TNF-α, IL-1β and IL-6 during Weeks 1 or 2 were used to determine the levels of adipokines in cows with metritis.

§Mean ± SEM serum concentrations of adiponectin, leptin, TNF-α, IL-1β and IL-6 during Week 5 were used to determine adipokine concentrations in cows with clinical endometritis, or subclinical endometritis, or normal cows.

^a-c^Within column, means without common superscript differ (*P* < 0.05).

*Controlling for body condition score categories [low (2 and 2.5) and high (3, 3.5 and 4)]; P < 0.05), weeks postpartum (P < 0.05), weeks postpartum by body condition score categories interaction (P < 0.05) and uterine inflammatory conditions by body condition score categories interaction (P < 0.05).

### Serum concentrations of adipokines, insulin, and IGF-1 in uterine inflammatory condition and body conditions score categories during peripartum

**Figure 1:**
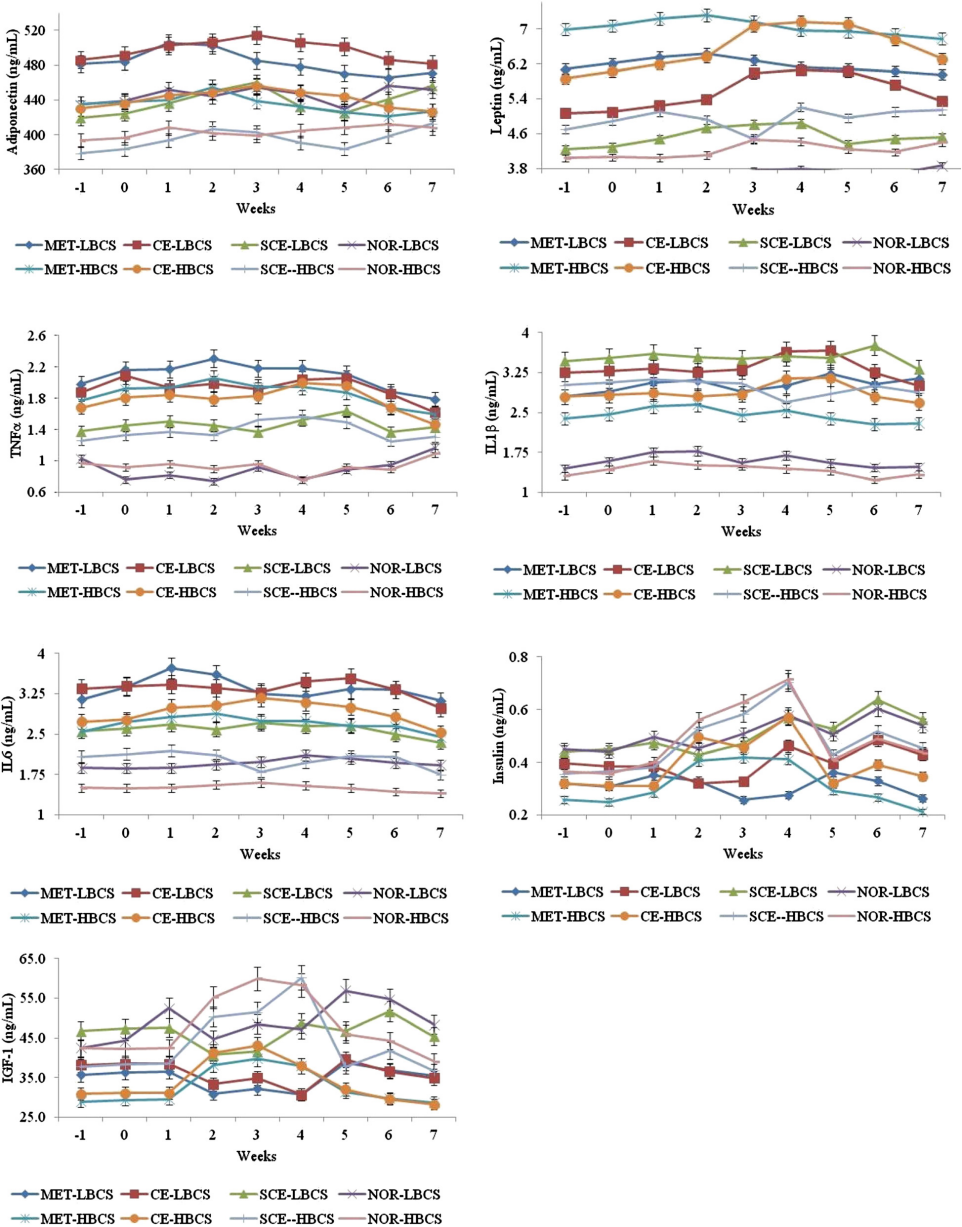
Mean ± SEM serum concentrations of adiponectin, leptin, tumor necrosis factor-α, interleukin 6, interleukin 1β, insulin, and insulin like growth factor-1 in uterine inflammatory condition categories* in dairy cows with low and high body condition‡ during peripartum. *Refer Table 1 for definition of uterine inflammatory condition; ‡Body condition score: from 1 (emaciated) to 5 (obese); Refer Table 4. Week 0: week of calving; MET: Metritis; CE: Clinical endometritis; SCE: Subclinical endometritis; NOR: Normal; LBCS – Lower (2 and 2.5) body condition score; HBCS: Higher (3 to 4) body condition score. Within a uterine inflammatory condition categories, means differ between weeks (P < 0.05). Within body condition score categories means differ between weeks (P < 0.05). Within a week, means differ between uterine inflammatory condition and body condition score categories (P < 0.05); Refer Table 2 for significant differences.

Differences in serum concentrations of adipokines, insulin, and IGF-1 (according to uterine inflammatory condition and body condition score categories) during the 7 weeks post calving are shown in Figure 1. Cows with metritis and clinical endometritis almost always had higher adipokines and lower metabolic hormone levels than subclinical endometritis and normal cows at any time-point during the study period, except for insulin and IGF-1. Similarly, cows with lower body condition score had higher adipokines and metabolic hormone levels than cows with higher body condition score. There were significant interactions for the weeks in postpartum by body condition score category, and for the uterine inflammatory condition by body condition score category and for the weeks in postpartum by the uterine inflammatory condition (P < 0.05).

### Serum concentrations of adipokines, insulin, and IGF-1 in persistent uterine inflammatory condition

Serum adipokine concentrations differed between persistent uterine inflammatory condition categories (persistently infected vs. normal and recovered cows), weeks postpartum [−1, 0, 1 to 7], and body condition scores [2 and 2.5 (low) vs. 3, 3.5 and 4 (high)] (Table 4; P < 0.05). There were significant interactions for the weeks in postpartum by body condition score category, persistent uterine inflammatory condition by weeks in postpartum and for the persistent uterine inflammatory condition by body condition score categories (Table 4; P < 0.05).

**Table 4:** ‘ *P *’ values* from repeated measures of ANOVA for the effects of persistent uterine inflammatory condition‡, body condition score categories [low (2 and 2.5) and high (3, 3.5 and 4)] and weeks postpartum on adipokines in postpartum dairy cows

**Adipokine**	**Effect**	**DF**	**F Value**	***P*** **> F**
*Adiponectin*	Persistent uterine inflammatory conditions	1	11.01	<0.001
	Body condition score categories	1	7.71	0.001
	Weeks in postpartum	8	13.27	0.01
*Leptin*	Persistent uterine inflammatory conditions	1	16.04	0.01
	Body condition score categories	1	8.65	0.01
	Weeks in postpartum	8	13.51	0.03
*TNF-α*	Persistent uterine inflammatory conditions	1	14.56	0.001
	Body condition score categories	1	8.44	0.009
	Weeks in postpartum	8	15.78	0.04
*IL-1β*	Persistent uterine inflammatory conditions	1	13.15	<0.001
	Body condition score categories	1	6.16	0.001
	Weeks in postpartum	8	16.73	0.02
*IL-6*	Persistent uterine inflammatory conditions	1	13.28	<0.001
	Body condition score categories	1	7.47	0.01
	Weeks in postpartum	8	13.11	0.01
*Insulin*	Persistent uterine inflammatory conditions	1	11.91	<0.01
	Body condition score categories	1	7.78	0.03
	Weeks in postpartum	8	16.48	0.02
*IGF-1*	Persistent uterine inflammatory conditions	1	16.18	<0.01
	Body condition score categories	1	8.49	0.01
	Weeks in postpartum	8	13.81	0.01

*Accounted for persistent uterine inflammatory condition by body condition score categories, persistent uterine inflammatory conditions by weeks in postpartum and body condition score categories by weeks in postpartum interactions, except for IGF-1 (P < 0.01); ‡A cow diagnosed with uterine disease at both initial and follow-up examinations was considered to have persistent inflammation.

### Serum concentrations of adipokines, insulin, and IGF-1 in uterine inflammatory condition and body conditions score categories during peripartum

**Figure 2:**
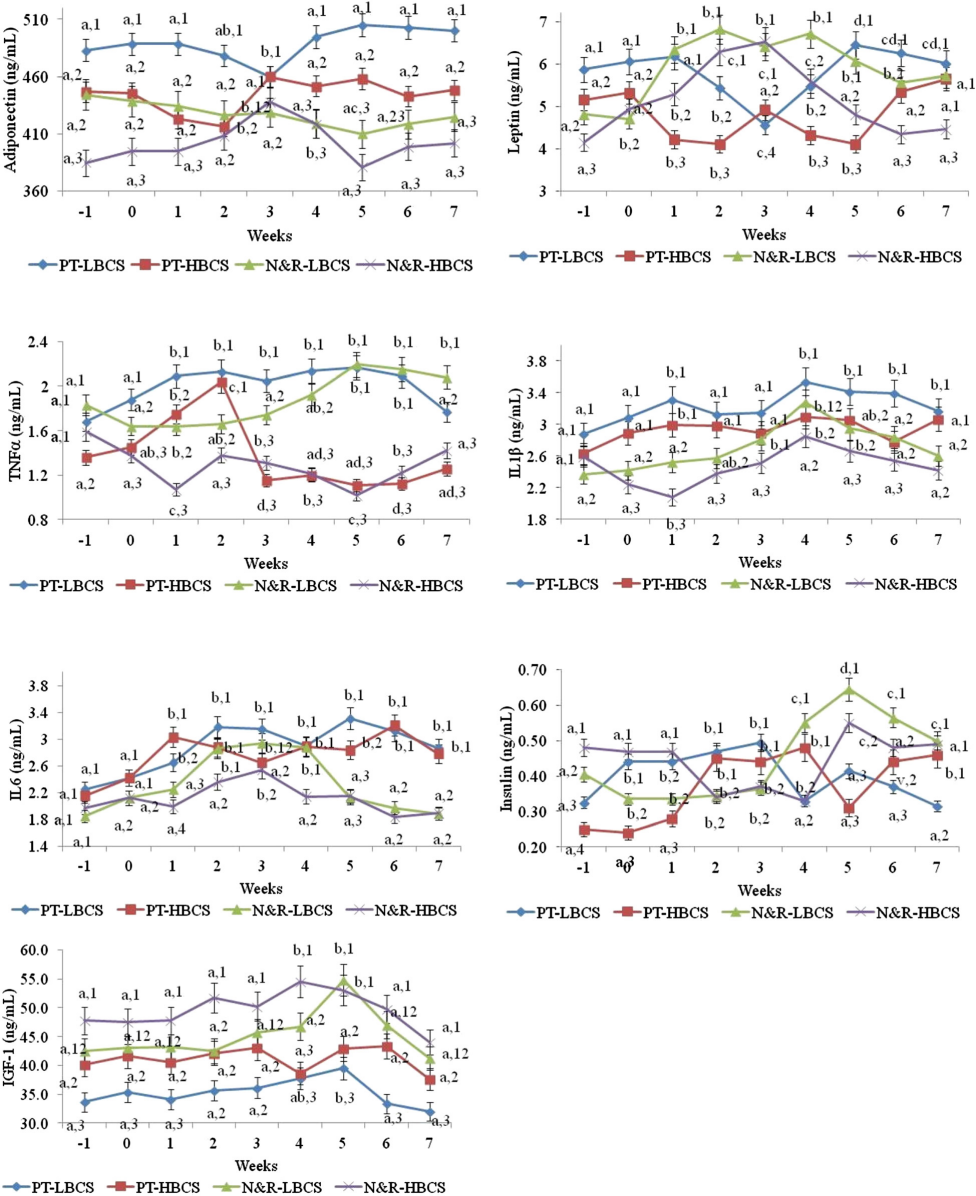
Mean ± SEM serum concentrations of adiponectin, leptin, tumor necrosis factor-α, interleukin 6, interleukin 1β, insulin, and insulin like growth factor-1 in persistent uterine inflammatory condition* categories in dairy cows with low and high body condition‡ during peripartum. *A cow diagnosed with uterine disease at both initial and follow-up examinations was considered to have persistent inflammation; ‡Body condition score: from 1 (emaciated) to 5 (obese). Week 0: week of calving; PT-LBCS: cows with persistent uterine inflammatory condition and lower (2 and 2.5) body condition score; PT-HBCS: cows with persistent uterine inflammatory condition and higher (3 to 4) body condition score; N&R-LBCS: normal and spontaneously recovered cows for persistent uterine inflammatory condition and lower (2 and 2.5) body condition score; N&R-HBCS: normal and spontaneously recovered cows for uterine inflammatory condition and higher (3 to 4) body condition score. ^a-d^Within a persistent uterine inflammatory condition and body condition score categories, means without a common superscript differ between weeks (P < 0.05). ^1-4^Within a week, means without a common superscript differ between persistent uterine inflammatory condition and body condition score categories (P < 0.05); (Also, refer Table 4 for significant differences).

Differences in mean serum concentrations of adipokines, insulin, and IGF-1 (according to persistent uterine inflammatory condition and body condition score categories) are shown in Figure 2. Cows with persistent uterine inflammatory condition almost always had higher adipokines and metabolic hormone levels than normal and recovered cows except for IGF-1. Similarly, cows with lower body condition score had higher adipokines and metabolic hormone levels than cows with higher body condition score. There were significant interactions for the weeks in postpartum by body condition score category, persistent uterine inflammatory condition by body condition score category and for the weeks in postpartum by the persistent uterine inflammatory condition (P < 0.05).

## Discussion

In this study, postpartum dairy cows diagnosed with metritis, clinical endometritis, or subclinical endometritis had higher serum concentrations of adiponectin, TNF-α, IL-1β and IL-6 compared to normal cows. In addition, adiponectin concentrations were higher for cows with low BCS compared to cows with high BCS throughout the study period. Cows with persistent uterine inflammation had higher serum concentrations of adiponectin, TNF-α, IL-1β and IL-6 compared to normal and recovered cows. Furthermore, cows with metritis or clinical endometritis had lower or lost body condition compared to those with subclinical endometritis or normal cows during the study period. Additionally, cows with persistent uterine inflammation lost body condition.

In the early stages of inflammation, blood concentrations of proinflammatory cytokines are increased [29]. Weight loss caused dramatic decreases in IL-6 and TNF-α and increased adiponectin [14,30]. The physiology of appetite and feeding behavior is very complex and involves neuroanatomical, neurophysiological and neurochemical pathways. Evidence suggests that cytokines IL-1, IL-6, TNF-α and interferon suppress appetite. Conditions such as inflammation, infection and cancer are usually associated with increased cytokine production, have also been associated with anorexia and weight loss [31]. In addition, in cows with lower body condition, it is reasonable to expect high adiponectin concentrations as a cause-effect relationship due to depletion of tissue fat depots. In the present study, there were higher concentrations of adiponectin, TNF-α, IL-1β and IL-6 in cows with uterine inflammatory conditions that also had lower BCS.

Circulating IL-6 concentrations were elevated in dairy cows before calving, but they decreased to baseline by 8 d after calving [32]. During the postpartum interval, IL-6 is expressed in the bovine endometrium in a time-related manner, with a significant peak on Day 17 postpartum, perhaps representing a mucosal immune response in the uterus [33]. In addition, Fischer et al. (2010) reported IL-6 mRNA expression was not influenced by inflammation between 21 and 27 d postpartum [34], and Galvão et al. (2011) reported increased IL-6 gene expression in the seventh week postpartum in cows with subclinical endometritis [35]. It should be noted that in acute inflammation there is an increase peripheral cytokines in association with increased tissue cytokine expression. Interestingly, in the present study, IL-6 increased during Weeks 1 and 2 in cows with metritis, and during Weeks 4 and 5 in cows with clinical and subclinical endometritis. Further, the IL-6 also increased in persistently infected cows and cows with lower BCS. It is noteworthy that IL-6 has lipolytic activity [36]. Perhaps increased IL-6 concentrations during disease conditions and during weight loss as caused lipolysis, which not only provided energy but also provided necessary adipokines to increase resistance to disease. In response, Toll-like receptors 2 and 4 in the adipocyte respond to bacterial pathogens [37,38]. Cows that recover from a negative energy balance are able to restore body condition and have a more functional immune system. In contrast, increased IL-6 concentrations immediately after calving in this and other studies [32-35], supported the notion that altered cytokines mediated body condition loss, and body condition loss mediated alterations in anti- and pro-inflammatory cytokines which acted serially to prolong uterine inflammation in dairy cows.

Pro-inflammatory cytokines act on several targets that may exacerbate body condition loss of immunologically challenged animals. Muscle protein degradation and lipolysis are mediated by IL-1β, IL-6, and TNF-α. Furthermore, IL-1β inhibits the anabolic effects of insulin on skeletal muscle [38]. Consequently, IL-1β, IL-6, and TNF-α are part of a network that links muscle protein degradation and lipolysis with hepatic acute phase protein synthesis [39]. This clearly represents an integrated host response to inflammatory stimuli. Furthermore, many of the metabolic effects of peripheral immunological stress are mediated by the actions of cytokines in the brain. In that regard, IL-1β, IL-6, and TNF-α from activated leukocytes suppress the CNS, decrease appetite and feed intake, thereby causing weight loss. IL-1β, IL-6, and TNF-α were elevated in persistently infected cows and in cows with low BCS.

Interleukin 1β is secreted by mononuclear cells, including monocytes and macrophages, in response to infections [40]. In the present study, serum concentrations were higher in cows with metritis, and clinical or subclinical endometritis compared to normal cows. It is known that IL-1β acts centrally to induce anorexia (by acting on neurotransmitters) and peripherally it inhibits gastric motility, gastric emptying, and gastric acid secretion [41]. It also causes alterations in the endocrine system, including corticotrophin-releasing factor, cholecystokinin, glucagon, and insulin [42]. These central and peripheral actions of leukocytic cytokines can decrease feed intake by more than 50% during the acute phase of the disease. Perhaps cytokine-mediated neuroendocrine changes have a direct effect in cows with metritis and overt systemic signs, and they have an indirect effect in cows with clinical or subclinical endometritis without systemic involvement.

The cytokine TNF-α is produced by immune, epithelial, glandular epithelial and endothelial cells in the stromal layer of the bovine uterus [43-45]; in addition to regulating immunologic, inflammatory or reparative responses, it also controls prostaglandin (PG) synthesis in the bovine endometrium [46]. In the present study, serum TNF-α concentrations were higher in subclinical cows compared to normal cows, and higher in cows with metritis or clinical endometritis cows compared to those with subclinical endometritis or normal cows. PGF metabolites were higher in cows with postpartum uterine disorders [47-49], possibly due to TNF-α controlled endometrial PG synthesis. Galvão et al. (2011) evaluated modulation of TNF-α gene expression during the postpartum period in dairy cows and reported that expression was lower in endometritis cows compared to healthy cows 1 week postpartum; furthermore, there was a trend for significant interaction between endometritis and weeks postpartum on TNF-α gene expression [35]. Perhaps cows in that study were undergoing body condition loss, which caused an adipocyte-induced increase in cytokines. In contrast, in the present study, TNF-α concentrations were decreased during first 5 weeks, but reached precalving concentrations during Weeks 6 and 7 in normal cows and those with subclinical endometritis, respectively, whereas TNF-α was increased during the first 3 weeks postcalving and reached precalving concentrations during Weeks 4, 5 and 6 in cows with metritis. Concentrations of TNF-α were reduced during Week 7 in cows with metritis or clinical endometritis.

Based on metabolic alterations triggered by acute inflammation, we inferred that circulating cytokines may induce weight loss. Several studies have shown an increase in adiponectin expression after weight loss [50,51]. Weight loss may increase adiponectin receptor 1 (adipoR1) expression [48]. The mRNA expression of adiponectin is negatively correlated with body mass index and expression of the pro-inflammatory cytokines IL-6 and TNF-α, demonstrating that postpartum weight loss is a clear shift in adipokine profile [50-53]. Cytokines rapidly produce several systemic effects if the infection becomes generalized and/or prolonged [54,55]. In hepatocytes, cytokines up-regulate production and release of acute phase proteins [55]. In addition, exogenous IL-1β and TNF-α did not induce anorexia individually, but did so when administered simultaneously [56-58] by causing neuroendocrine changes. Interestingly, the increased ACTH induced by lipopolysaccharide (LPS) or the combination of IL-1β and TNF-α, was inhibited by pretreatment with a monoclonal antibody to IL-6, suggesting the ACTH profile in serum induced by LPS is actually the collective result of at least three cytokines [56-58].

Leptin has significant influence on energy balance and both innate and adoptive immunity. Together with IL-1, IL-6 and TNF-α, leptin acts as acute phase reactant during inflammation [21-23]. In natural immunity, leptin stimulates chemotaxis, phagocytosis and release of oxygen radicals in PMNs. In adaptive immunity, leptin stimulates proliferation of native T cells and promotes the secretion of T helper 1 cytokines. Dairy cows experience dramatic changes in energy metabolism and feed intake during the periparturient period [21]. Feed intake is reduced in the last trimester of pregnancy, and particularly during the last 2 to 3 weeks prepartum despite growing energy needs for fetus and mammary development. Generally, leptin is unlikely to contribute to reduced appetite at that time because plasma concentration decreases during the last 1 to 2 weeks preceding parturition. It remains possible that changes in plasma leptin at earlier times during pregnancy modulate maternal food intake. After parturition, the lower concentration of plasma leptin could promote a faster increase in voluntary feed intake [21]. Studies have observed that the IGF-1 and leptin levels of metritis-affected cows tended to decline compared to normal cows, and remained very low for weeks during postpartum, and also their body condition loss was more severe [59]. In this study the leptin concentrations were high in both metritis and clinical endometritis cows whereas IGF-1 concentrations were lower in other cow groups. Higher leptin levels in metritis and clinical endometritis cows in this study possibly explain reduced feed intake and loss in body condition. It should also be noted that the leptin concentrations decreased in cows with persistent uterine inflammatory condition and increased in normal and recovered cows during 2 to 3 weeks. The leptin concentrations reversed, they were decreased in normal and recovered cows and increased in persistently inflamed cows starting from 4 weeks postcalving. However, the leptin levels were remained high from weeks 1 to 5 in cows with high BCS.

The concentration of insulin remained high in normal and subclinical endometritis cows compared to clinical endometritis and metritis cows. In normal and subclinical endometritis cows, the insulin concentrations started to increase around 4 weeks, peaked at 5 weeks postpartum and decreased abruptly during 6 and 7 weeks postpartum, even though the change in insulin concentrations in clinical endometritis followed similar pattern as in normal and subclinical endometritis cows the concentration was lower than the normal and subclinical endometritis cows. The insulin level was lower in the metritis cows compared to other groups. However the insulin concentration increased at 3 weeks postcalving to the level as clinical endometritis and decreased abruptly to a lower concentration at 7 weeks. Cows with metritis that had continuous hyperketonemia during postpartum, the insulin level was decreased at first week postcalving starting from day 2 and the level of insulin was increased at 4 to 5 weeks to same concentrations in metritis cows [59]. However in this study, the insulin concentration was decreased during 4 to 7 weeks post calving. It may be expected that hyperketonemic cows with severe clinical signs of uterine inflammatory conditions should have stronger evidence of insulin resistance as a result of an additional effect of cytokine production on insulin unresponsiveness. Severe inflammatory conditions like metritis and subclinical endometritis with intensive release of cytokines potentially further depress insulin secretion of pancreatic β-cells and whole-body insulin responsiveness in dairy cows. In the present study, cows with persistent uterine inflammation had lower insulin during the study period.

In the present study, concentrations of IGF-1 in normal and subclinical endometritis cows remained high, whereas in cows with clinical endometritis and metritis concentrations of IGF-1 were low. Additionally, cows with persistent uterine inflammation had lower IGF-1 during the study period. Therefore, the current study provided evidence for an ongoing inflammatory response in the uteri of cows with low IGF-1. An antagonistic relationship between the proinflammatory cytokines and IGF generally occurs during disease conditions [37]. It is plausible that poor energy status indicated by low leptin and IGF-1 may therefore inhibit the ability of the cow to raise an effective immune response to the bacterial challenge after calving and also delay the general repair process within the endometrium, thus prolonging uterine involution. In comparison, cows with a positive energy balance or those that recovered from their energy deficit 2 weeks after calving, as evidenced by the increase in circulating IGF-1 concentrations, undergo uterine involution at a more rapid rate. In support of this, IGF may antagonize proinflammatory activity by decreasing expression of the IL-receptor and *via* suppression of cytokine signaling proteins [37].

The adipokines, insulin and IGF-1 were different between persistent and normal recovered cows during the study period. The insulin and IGF-1 were remained increased in cows with high BCS except for weeks 2, 3 and 4. The leptin levels were remained high from weeks 1 to 5 in cows with high BCS. It should be noted that there were BCS by uterine inflammatory condition, BCS by uterine inflammatory condition persistence categories and BCS by weeks in postpartum interactions indicating that BCS changed during the study period and that the BCS influenced the adipokines and metabolic biomarkers studied.

Uterine infections increase expression of mRNA transcripts in the endometrium that encode molecules associated with inflammation, such as the cytokines IL-1β and IL-6, and the chemokine IL-8 [31]. The impact of Gram-negative infections on tissues and the immune defense against these bacteria are highly dependent on recognition of LPS by TLR4. The endometrial cells secrete cytokines and chemokines in response to the LPS of Gram-negative bacteria via TLR4/ MYD88-dependent pathways, and inhibition of MAPK3/1 counters the proinflammatory response [60]. Adipose tissue contributes up to 35% of the circulating IL-6 and is critical to counter endometrial damage caused by pathogens where endometrial production of cytokines is limited [61]. Impairment in endometrium cytokine production could be due to decreased production or failure of obtaining the necessary stimulation or the endometrium could produce inhibitors of proliferation [62-65]. In cows where endometrial production of cytokines is impaired, the cow might depend on cytokines from adipose tissue. However, postpartum dairy cows that have impairment in both endometrial and adipose tissue production of cytokines suffer from persistent uterine inflammatory condition (Figure 2).

## Conclusions

The present study demonstrated that circulating adipokines, insulin, and IGF-1 differed in cows with metritis, clinical endometritis or subclinical endometritis, in cows with persistent postpartum uterine inflammation and in cows with low versus high body condition. Cows with metritis and clinical endometritis had higher adipokines and lower metabolic hormone levels than subclinical endometritis and normal cows during the study period. Cows with persistent postpartum uterine inflammation had higher serum concentrations of TNF-α, IL-6, leptin, but lower insulin and IGF-1 compared to normal and spontaneously recovered cows and there was a temporal association observed during the study period. Adiponectin, IL-1β, IL-6, and TNF-α were elevated in cows with lower body condition. Body condition was lesser for cows with metritis or clinical endometritis compared to normal cows. Further, cows with persistent inflammation lost at least 0.5 body condition score compared to normal and/or spontaneously recovered cows. Therefore, we inferred that body condition loss mediated increases in anti- and pro-inflammatory cytokines and that increased anti- and pro-inflammatory cytokines mediated body condition loss may act serially to prolong persistence of uterine inflammation in dairy cows.

## Competing interests

The authors declare that they have no competing interests.

## Authors’ contributions

RKK initiated and designed the project, performed most of the experiments and wrote the manuscript. VRK helped with conducting and analyzing ELISA. JRO and EJJ helped in sample collection and analysis of the ELISA. DAM and JRK contributed to the design of the project and manuscript preparation. All authors read and approved the final manuscript version.
